# Author's Reply

**DOI:** 10.1371/journal.pmed.0030285

**Published:** 2006-06-27

**Authors:** Richard L Kravitz

**Affiliations:** **1**University of California DavisDavis, CaliforniaUnited States of America

In my section of the *PLoS Medicine* Debate entitled “What Are the Public Health Effects of Direct-to-Consumer Drug Advertising?” [ 1], I noted that a two-year moratorium on direct-to-consumer (DTC) advertising of new drugs could “avoid another Vioxx tragedy, in which drug marketing got well ahead of the science.” Contrary to Michael Heinley's complaint [ 2], I did not suggest that Merck acted improperly. That is a matter for the courts to decide. My only point was that obtaining more information on the risk:benefit ratio of Vioxx prior to mass marketing would have resulted in more informed prescribing.

From the time of its approval on May 21, 1999, until its voluntary withdrawal on September 30, 2004, Vioxx was prescribed to more than 80 million patients [ 3]. During much of that time, Merck conducted a vigorous and highly successful DTC marketing campaign. Whatever one believes about early signals of excess cardiovascular risk, by 2004 the results of the APPROVe trial had convinced everyone, including Merck, that Vioxx represented a potential threat to public health [ 4, 5]. In the meantime, hundreds of thousands of patients sought and received Vioxx prescriptions as a result of watching DTC advertisements on television, and up to 16 per 1,000 may have suffered untimely myocardial infarctions or strokes as a result [ 3]. If there had been a DTC advertising moratorium in place beginning in 1999, the science of adverse-event monitoring might have had a fighting chance to catch up.
